# Effectiveness of a School-Based Intervention to Improve Menstrual Hygiene Awareness and Access Among Adolescent Girls in Rural West Bengal

**DOI:** 10.7759/cureus.108123

**Published:** 2026-05-01

**Authors:** Moupriya Banerjee, Jayeeta Burman, Sembagamuthu Sembiah, Jeevanmuktha Somashekara

**Affiliations:** 1 Department of Community Medicine, Sarat Chandra Chattopadhyay Government Medical College & Hospital, Uluberia, IND; 2 Department of Community Medicine and Family Medicine, All India Institute of Medical Sciences, Kalyani, Saguna, IND

**Keywords:** accessibility, adolescent girls, menstrual hygiene, menstrual hygiene and sanitary napkin, school-based intervention, school health

## Abstract

Background

Menstrual hygiene management (MHM) remains a public health concern among adolescent girls in rural India. Although sanitary pad use has increased, gaps persist in hygienic practices and access. School-based interventions can address both knowledge and accessibility barriers. This study evaluated the effectiveness of a school-based intervention integrating menstrual hygiene education with subsidised sanitary pad distribution.

Methods

A single-group pre-post quasi-experimental study without a control group was conducted among 172 adolescent girls aged 12-17 years from three rural secondary schools in Howrah district, West Bengal. The intervention included a structured awareness session delivered by trained female teachers and the provision of subsidised sanitary pads to non-users over three months. Data were collected using a pre-tested structured questionnaire. Knowledge was assessed before and after the session, while hygiene practices and pad use were assessed at baseline and follow-up. Paired *t*-test and McNemar test were used for analysis.

Results

At baseline, 153 (89.0%) participants used sanitary pads; however, only 52 (30.2%) changed absorbents at recommended intervals, 65 (37.8%) practised adequate hygiene, and 68 (39.5%) followed safe disposal practices. Post-intervention, these improved to 145 (84.3%), 156 (90.7%), and 140 (81.4%), respectively (*p*<0.001). The mean knowledge score increased from 6.1 ± 2.0 to 11.4 ± 1.8 (*p*<0.001). Overall, sanitary pad use increased to 169 (98.3%) (*p*=0.001). Among baseline non-users (19, 11.0%), 16 (84.2%) adopted sanitary pad use following subsidised access.

Conclusion

This school-based intervention was associated with improvements in MHM, self-reported hygiene practices, and sanitary pad use. These findings suggest that integrated approaches addressing both behavioural and structural barriers may support better MHM in rural school settings.

## Introduction

Menstrual hygiene management (MHM) is a critical determinant of adolescent health, education and well-being. It refers to the use of safe menstrual materials, adequate water, sanitation and hygiene (WASH) facilities, and appropriate knowledge to manage menstruation in a hygienic and dignified manner. Inadequate MHM has been associated with reproductive and urogenital symptoms, psychosocial distress, stigma, restricted mobility and school absenteeism among adolescent girls, particularly in low- and middle-income countries [1]. Recent evidence also suggests that menstrual health challenges remain deeply shaped by structural barriers such as poverty, inadequate school sanitation and limited social support [2]. 

In India, progress in the use of hygienic menstrual methods has been documented, but important inequities persist. Analysis of National Family Health Survey (NFHS)-5 data among women aged 15-24 years showed that about 77% used hygienic menstrual absorbents, with substantially lower use in socioeconomically disadvantaged populations and in central and eastern India [3]. Rural adolescents continue to face lower access and poorer hygienic practices than their urban counterparts, indicating that product use alone does not ensure adequate menstrual hygiene management [4].

Beyond the use of sanitary pads, correct menstrual hygiene practices remain suboptimal. Recent Indian studies have shown persistent gaps in changing absorbents at recommended intervals, genital hygiene, safe disposal and pre-menarche awareness. A recent study from rural Bankura district, West Bengal, reported that 74.8% of girls lacked prior understanding of menstruation and that inadequate disposal practices and use of homemade reusable absorbents remained common, highlighting the continued local burden in eastern India [5].

The consequences of poor menstrual hygiene extend beyond discomfort. Menstrual health challenges have been linked to reduced school attendance and participation. A recent school-based study from North India reported that nearly one-third of adolescent girls were absent from school during menstruation, with barriers including pain, restrictions, fear of staining and difficulty changing absorbents in school. More broadly, improvements in school MHM facilities and practices have been associated with reduced absenteeism, underscoring the importance of supportive school environments alongside individual knowledge [6].

Socio-cultural factors play a significant role in shaping menstrual hygiene practices. In many communities, menstruation continues to be associated with stigma and restrictions, limiting open discussion and access to reliable information. Girls often experience embarrassment while purchasing sanitary products and may lack support from family members, particularly male members, in managing menstruation. These factors contribute to a persistent gap between awareness and practice, suggesting that knowledge alone may be insufficient to improve menstrual hygiene behaviours in the absence of supportive environments and access.

School-based interventions have emerged as an important platform for addressing menstrual hygiene challenges. Schools provide a structured environment where adolescents can receive accurate information and develop positive health behaviours. Recent evidence suggests that educational interventions can improve menstrual knowledge, but their impact may be limited when affordability, accessibility and school infrastructure are not addressed simultaneously. Quasi-experimental studies from India indicate that structured school-based menstrual health education can improve knowledge and hygiene-related outcomes, but most focus primarily on knowledge rather than the combined delivery of awareness and affordable product access within routine school systems [7-9].

Despite a growing literature on menstrual hygiene in India, the key gap is not the complete absence of studies, but the relative lack of implementation-focused evidence from routine rural school settings. Many published studies are cross-sectional, and among intervention studies, few have examined how a teacher-facilitated, school-based model that combines awareness-raising with access to subsidised sanitary products operates in practice. Evidence is especially limited from rural eastern India, where social norms, affordability barriers and school-level constraints may shape both uptake and sustainability.

Conceptually, this study was informed by a social-ecological perspective, which recognises that menstrual hygiene behaviours are influenced not only by individual knowledge, but also by interpersonal support, school systems, access to products, WASH infrastructure and prevailing social norms. Accordingly, the intervention sought to address behavioural, institutional and structural barriers through teacher-led education, school-based access to affordable sanitary pads and reinforcement within the school environment. In this context, the present study was undertaken to assess baseline menstrual hygiene awareness, practices, and barriers among adolescent girls in rural schools in Howrah district, to implement a teacher-facilitated intervention combining awareness-raising and subsidised sanitary pad distribution, and to evaluate changes in knowledge, practices, and uptake over a three-month follow-up period.

## Materials and methods

Study design and setting

This quasi-experimental, single-group pre-post (before-after) interventional study was conducted between October 2024 and September 2025 in three government secondary schools located in rural areas of Howrah district, West Bengal, India. A concurrent control group was not included due to the pragmatic, school-based implementation context; therefore, the study does not estimate a counterfactual and is intended to assess short-term changes within the intervention setting. Howrah district was selected purposively as it represents the field practice area of the study institution, facilitating the feasibility of implementation, supervision, and follow-up. The selected schools were typical of government rural secondary schools in the region, allowing assessment of the intervention in a real-world setting. Prior permission to conduct the study was obtained from the Institutional Ethics Committee of Sarat Chandra Chattopadhyay Medical College and Hospital (Memo No: SCCGMCU/507/24 dated 25.06.24) and the respective school authorities. This study is reported in accordance with the Transparent Reporting of Evaluations with Nonrandomized Designs (TREND) statement [10]. The completed checklist is provided in the appendix.

Study population

The study population consisted of adolescent girls studying in classes VIII, IX, and X. All girls aged 12-17 years who had attained menarche, were present on the day of data collection, and provided assent along with parental or guardian consent were included in the study. Girls who were unwilling to participate were excluded.

A complete enumeration approach was adopted in the selected schools, whereby all eligible adolescent girls available during the study period were invited to participate. As the study aimed to include the entire accessible population within the intervention setting, a formal a priori sample size calculation was not undertaken. A total of 172 eligible participants were enrolled in the study.

Intervention

The intervention comprised two components: (i) a structured menstrual hygiene awareness session and (ii) provision of access to subsidised sanitary pads.

Awareness Component

A structured, school-based awareness session of approximately 45-60 minutes was conducted for all participants. The session was delivered by trained female teachers using a standardised module developed by the study team based on national guidelines and existing educational materials, to ensure uniformity of content across schools.

The module included the following domains: (i) basic menstrual physiology and normalcy of menstruation, (ii) recommended hygiene practices including frequency of absorbent change (every four to six hours), genital hygiene, and handwashing, (iii) safe disposal methods, (iv) common myths and misconceptions, and (v) maintenance of routine daily activities during menstruation.

Interactive teaching methods were used, including visual aids (charts/flipbooks), group discussion, and question-and-answer sessions to encourage participation and clarify misconceptions. To reinforce learning, teachers conducted brief follow-up interactions (10-15 minutes) during routine school activities at least once per month over the three-month follow-up period.

Teachers underwent a structured one-hour training session conducted by the investigators prior to implementation. The training included familiarisation with the standardised module, demonstration of key messages, and guidance on facilitating interactive discussions. To ensure standardisation of delivery, teachers were provided with a structured session outline and a key message checklist.

Implementation fidelity was monitored through teacher-maintained session logs documenting session delivery and reinforcement activities, along with periodic supervisory visits by the study team to ensure adherence to the planned content and delivery approach. Teachers’ feedback was used to address implementation challenges and maintain consistency across sessions.

Access to Subsidised Sanitary Pads

The second component involved facilitating access to subsidised sanitary pads for participants who reported non-use due to financial constraints. Pads were procured in bulk from the Pradhan Mantri Bharatiya Jan Aushadhi Kendra supply at ₹20 per pack (≈six pads), compared with a local market price of approximately ₹50 per pack, to ensure affordability and continuous availability. A buffer stock was maintained at each school to prevent stock-outs.

Pads were provided to eligible participants at this subsidised price through designated female teachers. Distribution was on request, confidential, and carried out from a designated, discreet storage location within the school to ensure privacy and acceptability. Teachers maintained stock and issue registers documenting quantities dispensed and remaining stock.

To ensure an uninterrupted supply, the study team conducted monthly stock checks and replenishment based on consumption records. This teacher-facilitated distribution model, adapted from existing school-based practices, was used to enhance accessibility and acceptability within the school setting.

Implementation fidelity was monitored through periodic review of teacher-maintained registers and supervisory visits by the study team, ensuring adherence to the planned subsidy mechanism and distribution procedures.

The intervention was described in accordance with the Template for Intervention Description and Replication (TIDieR) checklist [11] to enhance reproducibility.

Study outcomes

The primary outcomes of the study were changes in menstrual hygiene knowledge and practices following the intervention. Knowledge regarding menstruation was assessed using seven items, each scored as correct or incorrect. A composite knowledge score was calculated by assigning one point to each correct response, yielding a total score of zero to seven for each participant. In addition, individual knowledge components were analysed separately.

Menstrual hygiene practices were assessed using a structured questionnaire covering key domains, including type of absorbent used, frequency of changing absorbent, genital hygiene practices, and method of disposal.

For analytical purposes, each component was categorised as appropriate or inappropriate according to standard recommendations and the existing literature.

Use of sanitary pads was considered appropriate, while use of cloth or other materials was classified as unsanitary. Changing absorbents every four to six hours was considered appropriate. Genital hygiene was considered appropriate if participants reported cleaning the genital area during menstruation, with water and soap considered optimal. Disposal practices were classified as safe if used absorbents were wrapped and disposed in a bin or burned, and unsafe if discarded in open areas or gutters.

Each practice component was analysed separately to assess changes between baseline and follow-up. Improvement in menstrual hygiene practices was evaluated by comparing responses related to key behavioural indicators, including frequency of absorbent change, genital hygiene, and disposal practices, after a follow-up period of three months.

Secondary outcomes included uptake of sanitary pads among baseline non-users. This was assessed as the proportion of participants who were not using sanitary pads at baseline but adopted their use during the follow-up period after being provided access to subsidised pads.

Data collection

Data were collected using a pre-tested, structured questionnaire developed based on existing literature and standard menstrual hygiene guidelines (appendix). The questionnaire included sections on socio-demographic characteristics, menstrual history, hygiene practices, and knowledge regarding menstruation. It was initially prepared in English, translated into Bengali, and back-translated to ensure accuracy. Content validity was ensured through review by subject experts in community medicine and public health, who assessed the questionnaire for relevance, clarity, and comprehensiveness. The tool was pre-tested among adolescent girls in a similar setting outside the study area, and necessary modifications were made to improve clarity and cultural appropriateness.

As the questionnaire predominantly comprised categorical items assessing distinct domains, internal consistency measures such as Cronbach’s alpha were not uniformly applicable.

To minimise social desirability bias, data collection was conducted in a confidential setting, and participants were assured of anonymity and that their responses would not affect their access to services.

Data analysis

Data were analysed using IBM SPSS Statistics for Windows, Version 26 (Released 2019; IBM Corp., Armonk, New York, United States). Continuous variables were summarised as mean and standard deviation, and categorical variables as frequencies and percentages.

The composite knowledge score (range: 0-7) was treated as a continuous variable. Pre- and post-intervention knowledge scores were compared using the paired t-test. Effect size was expressed as mean difference (MD) with 95% confidence interval (CI), and Cohen’s d was calculated to assess the magnitude of change.

Categorical variables related to knowledge and menstrual hygiene practices were compared between baseline and follow-up using the McNemar test. Effect sizes for categorical variables were expressed as risk differences (RDs) with 95% confidence intervals.

All tests were two-tailed, and a p-value of <0.05 was considered statistically significant. Only participants with complete pre- and post-intervention data were included in the analysis.

## Results

The majority of participants were aged 15-16 years (n=78, 45.3%), followed by 13-14 years (n=63, 36.6%) and 17-18 years (n=31, 18.0%). Participants were almost equally distributed across classes VIII (n=58, 33.7%), IX (n=57, 33.1%), and X (n=57, 33.1%). Most belonged to nuclear families (n=101, 58.7%), and mothers had at least a primary education in most cases. More than half of the participants (n=98, 57.0%) reported that they were not aware of menstruation before menarche. The most common source of first information was the mother (n=89, 51.7%), followed by peers (n=47, 27.3%) and teachers (n=24, 14.0%). At baseline, 153 (89.0%) participants were using sanitary pads, while 19 (11.0%) used cloth or other materials (Table 1).

**Table 1:** Changes in the knowledge outcomes and the composite knowledge score among all participants (n=172) RD: risk difference; MD: mean difference; CI: Confidence interval.

Variable	Pre-test	Post-test	Effect size (95% CI)	p-value
Knowledge score (0–7)	4.1 ± 2.0	5.0 ± 1.8	MD = 0.9 (0.62–1.18); Cohen’s d = 0.47	<0.001
Menstruation is normal	172 (100.0)	172 (100.0)	—	—
Organ responsible (uterus)	153 (88.9)	168 (97.7)	RD = 0.087 (0.035–0.139)	<0.001
Correct age of menarche	138 (80.2)	155 (90.1)	RD = 0.099 (0.040–0.158)	<0.001
Absorbents should be changed regularly	85 (49.4)	149 (86.6)	RD = 0.372 (0.281–0.463)	<0.001
Genital hygiene is important	112 (65.1)	164 (95.3)	RD = 0.302 (0.227–0.377)	<0.001
Safe disposal awareness	106 (61.6)	145 (84.3)	RD = 0.227 (0.145–0.309)	<0.001
Misconception corrected	150 (87.2)	167 (97.1)	RD = 0.099 (0.050–0.148)	<0.001

The mean knowledge score increased from 4.1 ± 2.0 to 5.0 ± 1.8, with a moderate effect size (Table 1). Improvements were observed across all knowledge items. Awareness of the organ responsible for menstruation increased from 88.9% to 97.7%, and knowledge of the correct age of menarche improved from 80.2% to 90.1%. Larger improvements were noted in knowledge about absorbent changes (from 49.4% to 86.6%) and genital hygiene (from 65.1% to 95.3%). Misconceptions regarding the restriction of physical activity decreased (Table 2).

**Table 2:** Changes in menstrual hygiene practices among participants (n=172) RD: risk difference; CI: confidence interval.

Practice	Pre-test n (%)	Post-test n (%)	Effect size (95% CI)	p-value
Use of sanitary pads	153 (89.0)	169 (98.3)	RD = 0.093 (0.047–0.139)	0.001
Changing every 4–6 hours	52 (30.2)	145 (84.3)	RD = 0.541 (0.468–0.614)	<0.001
Cleaning genital area	65 (37.8)	156 (90.7)	RD = 0.529 (0.455–0.603)	<0.001
Safe disposal	68 (39.5)	140 (81.4)	RD = 0.419 (0.346–0.492)	<0.001

All menstrual hygiene practices improved following the intervention (Table 2). The use of sanitary pads increased from 89.0% to 98.3%. The proportion of participants changing absorbents at recommended intervals improved from 30.2% to 84.3%, while genital hygiene practices improved from 37.8% to 90.7%. Safe disposal practices also increased from 39.5% to 81.4% (Table 2).

Among the 19 (11.0%) participants who were not using sanitary pads at baseline, subsidised sanitary pads were made available through the intervention. During the follow-up period, 16 (84.2%) participants adopted sanitary pad use, while three (15.8%) did not utilise the service.

## Discussion

The present study found that a school-based intervention combining menstrual hygiene education with access to subsidised sanitary pads was associated with improvements in knowledge and selected self-reported hygiene practices among adolescent girls. Given the single-group pre-post design, these findings should be interpreted as associations rather than causal effects.

The high baseline use of sanitary pads observed in this study reflects the changing pattern of menstrual hygiene practices in India. Earlier studies, such as those by Kansal et al. and Sarkar et al., reported much lower pad usage (11% and 31%, respectively), indicating significant progress over time [12,13]. However, similar to the present findings, studies by Borkar et al. and Boruah et al. have shown that, even with higher levels of pad use (around 70-75%), appropriate hygiene practices remain inadequate [14,15]. This suggests that product access alone may be insufficient to ensure appropriate MHM.

The observed improvements in knowledge and practices are consistent with findings from educational intervention studies. Vagha et al. reported significant improvements in menstrual hygiene awareness and practices following structured school-based education [16]. However, unlike many such studies, the present intervention combined education with facilitated access to subsidised products within the school, which may help explain the observed improvements in behavioural outcomes.

The mechanisms underlying the observed improvements are likely multifactorial. Although baseline awareness of menstrual hygiene and sanitary pad use was relatively high, important gaps existed in practice-specific knowledge, particularly regarding frequency of absorbent change, genital hygiene, and safe disposal. The structured awareness sessions may have addressed these gaps, leading to improvements in correct practices rather than basic awareness.

In addition, the provision of subsidised sanitary pads within the school setting may have reduced barriers related to affordability and access, enabling adoption of recommended practices. The involvement of teachers likely contributed to reinforcement of key messages, improved acceptability, and ease of access, thereby facilitating behaviour change. These findings suggest that behavioural improvements were driven by the interaction of knowledge refinement, improved access, and supportive school-based delivery mechanisms. These findings are consistent with a social-ecological understanding of menstrual hygiene, where behaviours are shaped by both individual and contextual factors. 

A key finding of this study is the high uptake of subsidised sanitary pads among baseline non-users (84.2%). This supports the role of affordability and accessibility in influencing menstrual product use. Similar observations have been reported in Indian studies evaluating government and community-based pad distribution programmes. Surendran et al. found that the utilisation of government-supplied pads was limited by factors such as supply constraints and limited awareness [17]. In contrast, the school-based, teacher-facilitated distribution model used in this study may have reduced these barriers by ensuring consistent availability, privacy, and convenience. 

Interventional studies further support the importance of product access. Shah et al. reported that after an intervention in rural Gujarat, the use of unhygienic absorbents declined substantially, although the adoption of sanitary pads was influenced by affordability and acceptability [7]. Similarly, Vayeda et al. demonstrated that integrated interventions increased the use of safe menstrual absorbents from 69.0% to 90.5% among adolescent girls [18]. Programme-level evidence from Achuthan et al. also showed that menstrual hygiene schemes increased sanitary pad use and hygienic practices in rural populations [6].

These findings also align with programme-level experiences from school-based menstrual hygiene initiatives (e.g., Khushi scheme), where improvements in access were observed, but operational challenges, including supply consistency, persisted [19]. In this context, the present study suggests that institution-based delivery models may help strengthen last-mile access within existing programme frameworks.

From a programmatic perspective, the intervention components used in this study - teacher-led education, school-based product access, and reinforcement - are broadly compatible with existing Indian platforms such as the Rashtriya Kishor Swasthya Karyakram (RKSK) [20] and the School Health Programme under Ayushman Bharat [21]. While the present study was not designed to evaluate scalability, such approaches may be feasible to integrate within existing school health activities, subject to local adaptation and resource availability.

The cost implications of the intervention were not formally evaluated; however, the use of low-cost sanitary pads (e.g., from Jan Aushadhi Kendra) and teacher-led delivery suggest a potentially low-cost, resource-efficient model. Future studies should incorporate formal cost and cost-effectiveness analyses to inform programmatic decision-making.

Overall, the findings indicate that menstrual hygiene interventions in school settings may benefit from integrated approaches that address both behavioural and structural barriers. However, given the study design, these observations should be interpreted cautiously, and further research is required to establish effectiveness and sustainability.

Limitations

The quasi-experimental single-group pre-post design without a control group limits causal inference and is susceptible to internal validity threats. Observed improvements may partly reflect a Hawthorne effect (behaviour change due to observation) and a testing effect (influence of repeated assessments). No adjustment for potential confounders was undertaken, and unmeasured factors such as concurrent information exposure or peer influence may have contributed to the findings.

The study was conducted in a limited number of schools in a single rural district, which may affect generalisability. No formal sample size calculation was undertaken; therefore, the study may not have been adequately powered for all outcomes, and the findings should be interpreted as exploratory. Menstrual hygiene practices were self-reported and may be subject to recall and social desirability bias. The three-month follow-up may also not reflect the long-term sustainability of the observed changes. Subgroup analyses (e.g., by age or baseline sanitary pad use) were not undertaken, which may limit understanding of differential effects across participant groups.

## Conclusions

A teacher-facilitated, school-based intervention that combined menstrual hygiene awareness with access to affordable sanitary products was associated with improvements in knowledge and selected self-reported hygiene practices among adolescent girls. The findings suggest that, even in settings with relatively high baseline use of sanitary pads, gaps may persist in appropriate hygiene practices, including frequency of change and safe disposal.

Based on these findings, improvements in menstrual hygiene practices in similar school settings may be supported by integrating structured, curriculum-based education with teacher involvement in information delivery and in facilitating access to affordable menstrual products, as well as ensuring their consistent availability. Strengthening supportive school environments, including adequate WASH facilities and efforts to reduce stigma, may further facilitate adoption of recommended practices. Incorporating simple monitoring and reinforcement within routine school activities may help sustain these improvements over time. Further studies with comparative designs and longer follow-up are needed to better understand the effectiveness and scalability of such approaches across different settings.

## Appendices

**Table 3:** Study questionnaire

S. No.	Question	Response Options
Sociodemographic Information		
1	Age (years)	____
2	Class	VIII / IX / X
3	Type of family	Nuclear / Joint
4	Mother’s education	Illiterate / Primary / Secondary / Higher
5	Monthly family income (₹)	<10,000 / 10,000–20,000 / >20,000
Menstrual History		
6	Age at menarche (years)	<12 / 12–13 / >13
7	Were you aware of menstruation before menarche?	Yes / No
8	Source of first information	Mother / Teacher / Friends / Others
Menstrual Hygiene Practices		
9	Absorbent used during menstruation	Sanitary pads / Cloth / Others
10	Frequency of changing absorbent	<4 hours / 4–6 hours / >6 hours
11	Do you clean genital area during menstruation?	Yes / No
12	Method of cleaning	Water only / Water and soap
13	Method of disposal	Wrap & bin / Open disposal / Burn / Others
Knowledge Assessment		
14	Menstruation is a normal physiological process	Yes / No
15	Organ responsible for menstruation	Uterus / Stomach / Don’t know
16	Normal age of menarche	10–14 years / Other
17	Absorbents should be changed regularly	Yes / No
18	Genital hygiene is important during menstruation	Yes / No
19	Used pads should be disposed safely	Yes / No
20	Girls should avoid physical activity during menstruation	Yes / No
Follow-up (Acceptability)		
21	Did you attend awareness session?	Yes / No
22	Was the session helpful?	Yes / No
23	Did you use subsidised sanitary pads?	Yes / No
24	Was access easy?	Yes / No
25	Will you continue using sanitary pads?	Yes / No
